# The role of miR-29c/B7-H3 axis in children with allergic asthma

**DOI:** 10.1186/s12967-018-1590-8

**Published:** 2018-08-03

**Authors:** Xinxing Zhang, Xin Zhao, Huiming Sun, Yongdong Yan, Li Huang, Wenjin Gu, Wujun Jiang, Yuqing Wang, Canhong Zhu, Wei Ji, Chuangli Hao, Zhengrong Chen

**Affiliations:** 1 Department of Respiratory Disease, Children’s Hospital of Soochow University, Soochow University, Suzhou, China; 2 Department of General Surgery, The First Affiliated Hospital of Soochow University, Soochow University, Suzhou, China

**Keywords:** Asthma, Children, MircoRNA, B7-H3, Th cell differentiation

## Abstract

**Background:**

MicroRNAs play roles in the pathogenesis of bronchial asthma. However, the mechanism of miR-29c in allergic asthma remains unclear. This study is to elucidate the regulation of Th cell differentiation by miR-29c in mononuclear macrophages.

**Methods:**

A total of 52 children with asthma exacerbation and 26 children as controls were enrolled in the study. CD14^+^ monocytes were isolated from the peripheral blood. Differential expressions of microRNAs were evaluated using microarray analysis and miR-29c expression in monocytes was determined by qRT-PCR. The plasma B7-H3 was determined by ELISA. Transfection studies and luciferase reporter assay were performed to confirm target gene of miR-29c and its function.

**Results:**

Compared to controls, 88 miRNAs in blood monocytes were up-regulated and 41 miRNAs down-regulated including miR-29c in asthma children. Children with asthma exacerbation had significantly lower level of miR-29c and higher level of plasma B7-H3 compared to controls (both P < 0.05). Functional studies based on luciferase reporter assay and immunofluorescence staining suggest that B7-H3 is the direct target of miR-29c and transfection anti-miR-29c into macrophages could enhance ROR-γt and GATA-3 expression in co-cultured CD4^+^ T cells and increase levels of IL-4 and IL-17 in supernatants.

**Conclusion:**

The axis of miR-29c/B7-H3 plays an important role in children with asthma through regulating Th2/Th17 cell differentiation and may provide new targets for treatment of asthma.

## Background

Allergic asthma is a chronic inflammatory disorder of the airways characterized by airway hyper-responsiveness (AHR), eosinophil recruitment, mucus hypersecretion, and irreversible remodeling of the airways. Many studies dedicated that T-helper type 2 lymphocytes (Th2 cells) or Th17 cells play a vital role in allergic asthma via interleukin (IL)-4, IL-5, IL-13, IL-17 secretion which are associated with AHR and inflammatory cells infiltration [1–4]. Although these cytokines are hallmarks of Th2 immunity observed in models of allergic asthma, the molecular mechanisms leading to this abnormal Th2 response remain largely unclear.

Th activation and differentiation is associated with costimulatory molecules expressed on antigen presenting cell (APC) including B7 family molecules. Our previous study showed that blockage of B7-H3 could alleviate the asthmatic syndrome, decreased the levels of B7-H3 positive cells in the lung tissues, abrogated hypercellularity, eosinophil infiltration, and mucus production, and inhibited IL-4 and IL-17 production in bronchoalveolar lavage fluid [5]. Recently, our study demonstrated that B7-H3 could induce GATA-3 and ROR-γt expression in human naïve CD4^+^ T cells and enhance IL-4 and IL-17 secretion in vitro [6]. Taken together, these studies indicated that B7-H3 plays an important role in immunopathogenesis of asthma through regulating of Th2 and Th17 differentiation. However, the mechanism of how to regulating B7-H3 have not been fully studied.

MicroRNAs (miRNAs) are short non-coding RNAs that function as post-transcriptional modulators of gene expression by either promoting mRNA degradation or blocking protein translation [7, 8]. The miRNAs could control differentiation and function, and thus regulate inflammation of host immune cells, especially for APC and T cells [9, 10]. Recent studies demonstrated that several miRNAs take part in allergic airway inflammation via regulating Th2 differentiation such as miR-21 [11], miR-126 [12] and miR-145 [13]. miR-187 could inhibit cell growth and migration in clear cell renal cell carcinoma though targeting B7-H3 [14]. miR-29c-3p (miR-29c) plays an important role in mediating progression of cutaneous melanoma through regulating B7-H3 [15].

However, to our knowledge, there was no report about miR-29c in inflammatory disease such as asthma. Thus, this study is to better characterize the specific role of miR-29c targeting B7-H3 in asthma.

## Methods

### Patients

Hospitalized children with asthma exacerbation without treatment prior to admission were enrolled in this study from Children’s Hospital of Soochow University. The diagnosis of asthma and its severity of asthma exacerbation were confirmed according to Global Initiative for Asthma guidelines (http://ginasthma.org/). Exacerbation is an acute or sub-acute worsening in symptoms and lung function from the patients’ usual status. Upon hospital admission of the patients, pediatricians completed a questionnaire regarding demographic and clinical data of the patients. Meanwhile, peripheral blood samples were obtained for miR-29c and plasma B7-H3 detection before treatment with systemic steroids (acute phase) and before discharge (convalescent phase). The age-matched controls were chosen randomly from surgery wards without history of lung disease, wheezing, allergy, AHR and respiratory infections within 4 weeks. This study was conducted with the approval of the Institutional Human Ethical Committee of Children’s Hospital of Soochow University. An informed consent was obtained from all the subjects or guardians who participated in this study and all methods were performed in accordance with the relevant guidelines and regulations.

### Isolation of CD14^+^ monocytes from peripheral blood

Peripheral blood (2 ml) were obtained using heparin-natrium tube from all asthmatic children and controls and then peripheral blood cells (PBMCs) were isolated by Ficoll–Hypaque gradient centrifugation. Primary monocytes were isolated from PBMCs using a negative selection kit (MiltenyiBiotec, BergischGladbach, Germany) and further purified with a CD14^+^ selection kit (StemCell Technologies, Vancouver, British Columbia, Canada). The purity of the monocyte preparation was > 95% as identified by anti-CD14 staining and the cell concentration is range ~ 10^5^ to 10^6^/ml.

### Microarray analysis of miRNAs

Pooled CD14^+^ monocytes from three asthmatic children and three controls were used for miRNA microarray analysis. Total miRNA from these three pooled samples was extracted as previously described [16]. Microarray hybridization, data generation, and normalization were performed by KANGCHEN Biological Engineering Co. Ltd using human miRNA chip (miRCURY™, Exiqon, Denmark) which contain probes for 3100 miRNAs. Differentially expressed miRNAs were designated as up-regulated or down-regulated more than 2-fold in asthmatic children compared to controls.

### Luciferase reporter assay

Oligonucleotides corresponding to the miR-29c binding site in the B7-H3-3′UTR or a single-base mutant were synthesized and inserted into the XbaI site immediately downstream from the stop codon of firefly luciferase of the pGL3-control vector (Novobio Co. Ltd, Shanghai, China). Human monocyte cell line THP-1 was obtained from American Type Culture Collection (ATCC, Manassas, VA, USA). THP-1 cell were co-transfected in 24-well plates using Lipofectamine 2000 reagent (Invitrogen) according to the manufacturer’s protocol, with 50 ng of the firefly luciferase reporter, 1 ng of the renilla luciferase reporter (Promega) as transfection control, and 100 nM pLenti-miR-29c (Novobio Co. Ltd, Shanghai, China). Firefly and renilla luciferase activities were measured sequentially using dual-luciferase assays (Promega) 24 h after the transfection and evaluated by the BioTek™ Microplate Reader.

### Transfection of miR-29c and B7-H3 detection by immunofluorescence staining

THP-1 cells were seeded at 1 × 10^5^ cells/60 mm dishes and then transfected with 100 nM pLenti-miR-29c, anti-miR-29c or empty vector using the Jetprime™ Transfection Reagent (VWR International, Radnor, PA). After transfection (24 h), cells were treated with 1 mg/ml Pronase E (E. Merck, Darmstadt, Germany) for 30 min at 37 °C to strip off B7-H3 protein already on the cell surface, and another 48 h later newly expressed B7-H3 protein level were measured by anti-B7-H3 mAb (8H9) immunofluorescence staining. Slides were imaged using a digital slide scanner and grey levels of slides were obtained using Image-Pro Plus software.

### Function of miR-29c on macrophage in regulating Th cell differentiation

1 × 10^5^/ml of THP-1 cells were cultured in RPMI 1640 medium (Life Technologies, Carlsbad, CA, USA) primed with 100 nM PMA (Sigma, Louis, MO, USA) for 48 h to induce macrophage differentiation. Then, 100 nM pLenti-miR-29c or anti-miR-29c was transfected into macrophage and cultured for another 48 h. After transfection, the harvested macrophages were co-cultured with CD4^+^ T cells (1 × 10^5^ cells/ml) which were isolated from peripheral blood of healthy persons (donate from Red Cross, Suzhou, China). After a 24 h culture at 37 °C, 5% CO_2_, proliferated T cells were harvested and seeded into 6-well flat-plate that was pre-coated with anti-CD3 mAb (50 ng/ml) and anti-CD28 mAb (500 ng/l). After another 24 h, cell-free supernatants were collected to measure cytokines of IL-4 and IL-17 (pg/ml) using ELISA Kits while cells were collected to measure the relative expression of mRNA of GATA-3 and ROR-γt as previously described [6].

### Effect of anti-B7-H3 mAb on Th cell differentiation co-cultured with macrophages transfected by anti-miR-29c

Macrophages transfected with anti-miR-29c were harvested and were co-cultured with CD4^+^ T cells (1 × 10^5^ cells/ml) in the presence of control IgG (20 µg/ml) or anti-B7-H3 mAbs (20 µg/ml). The specific procedures were same as above. IL-4 and Il-17 in supernatant were detected using ELISA while GATA-3 and ROR-γt were detected using qPCR.

### Real-time quantitative PCR (qRT-PCR) for miR-29c in hospitalized children with asthma exacerbation

According to the manufacturer’s protocol, total RNA was extracted from CD14^+^ monocytes using Trizol reagent (Invitrogen). Initially, 10 ng of total RNA was subjected to reverse transcription polymerase chain reaction using the TaqMan MicroRNA Reverse Transcription kit (Applied Biosystems) according to manufacturer’s protocol. The thermocycling conditions were: 30 min at 16 °C, followed by 30 min at 42 °C, 5 min at 85 °C and 5 min at 4 °C. qRT-PCR was performed using TaqMan Universal PCR Master Mix Kit (Applied Biosystems) in a Bio-Rad iQ5 Real-Time PCRSystem and U6 was used as an endogenous control. The reaction was performed in triplicate according to manufacturer’s protocol. The thermocycling conditions were: 50 °C for 2 min, 95 °C for 10 min, and 40 cycles of 15 s at 95 °C, followed by 1 min at 60 °C. After finalization of the qRT-PCR experiments, the average values of the cycle threshold (Ct) of the reactions in triplicate were determined. Data analysis was performed using the 2^−∆∆^Ct method.

### Plasma B7-H3 detection by ELISA

Peripheral blood samples were obtained from hospitalized asthmatic children and controls. The plasma was collected after centrifuged at 3000×*g* for 10 min at 4 °C. All samples of plasma were preserved at − 80 °C for subsequent assay of plasma B7-H3 by ELISA as previously described [17].

### Statistical analysis

The analyses were performed using the Statistical Package for SPSS for windows, version 17.0. The data are presented as mean ± STD. Student T-test were performed for the comparisons between asthmatic children and controls. As for experiments in vitro, one-way ANOVA was performed among different groups. Pearson’s correlation test was used to assess the correlations between miR-29c and plasma B7-H3. Differences between groups were considered statistically significant when P < 0.05.

## Results

### Demographic data and clinical characteristics of children with asthma exacerbation

Total of 52 children with allergic asthma exacerbation and 26 control children with selective surgery were enrolled in this study. The demographic data and clinical characteristics of children with asthma exacerbation are shown in Table 1. All the children with asthma had positive inhaled allergen and the average age is 5.2 ± 3.0 years.

**Table 1:** Demographic and clinical characteristics of children with asthma exacerbation

Parameters	Asthma exacerbation subjects (n = 52)
Age, years	5.2 ± 3.0
Male, n (%)	33 (63.5)
Family history of asthma or atopy, n (%)	30 (57.7)
History of other atopic disease, n (%)	36 (69.2)
Regular ICS treatment before, n (%)	25 (48.1)
Exacerbation severity	
Mild to moderate, n (%)	34 (65.4)
Severe, n (%)	18 (34.6)
Inhaled allergen, n (%)	52 (100)
Eosinophil count, × 10^9^/L	328 ± 255
CD19^+^CD23^+^, %	10.8 ± 4.9

### miRNAs are differentially expressed in asthma and control children

**Fig. 1:**
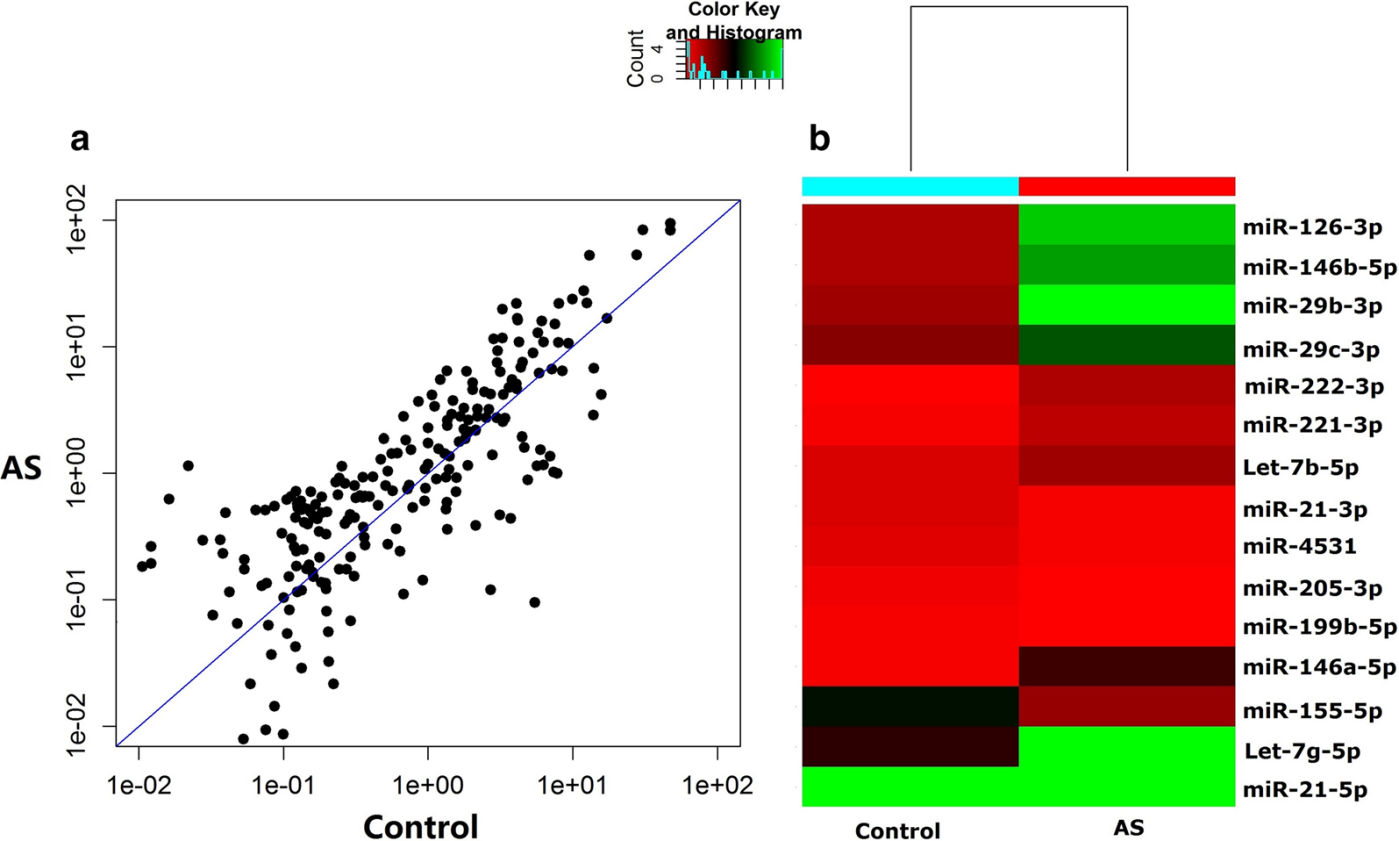
Microarray analysis of three children with asthma exacerbation and three controls. **a** Scatter plot of differentially expressed miRNAs determined by miRNA microarray analysis. **b** Heat map of 15 representative miRNAs including miR-29c

Our previous study demonstrated that B7-H3 mainly presents in CD14^+^ monocytes of peripheral blood [18]. To screen for differentially expressed miRNAs from CD14^+^ monocytes of asthmatic children and controls, miRNA microarrays were used to evaluate between these groups. As shown in Fig. 1a, b, a scatter plot and heat map were generated and 15 representative miRNAs had the binding sites to B7-H3 predicted by Target Scan 7.1 software. Compared to controls, there are 129 differentially expressed miRNAs of > 2-fold in asthmatic children. Of all differentially expressed miRNAs, 88 miRNAs were up-regulated and 41 miRNAs down-regulated (Table 2). Based on previous findings we have chosen to further study the effects of miR-29c in asthma andmiR-29c is one of all down-regulated miRNAs. It is verified by qPCR that the level of miR-29c in three asthmatic sample was lower than that of controls (0.4 ± 0.005 vs. 1.0 ± 0.003, P < 0.001).

**Table 2:** Differences in miRNA expression between children with asthma exacerbation and controls

Up-regulation				Down-regulation	
miRNAs	Fold	miRNAs	Fold	miRNAs	Fold
hsa-miR-23a-5p	52.52	hsa-miR-4533	4.63	hsa-miR-29c-3p	0.35
hsa-miR-4475	3.48	hsa-miR-1260	2.67	hsa-miR-12-5p	0.16
hsa-miR-4726-5p	2.57	hsa-miR-664b-3p	2.01	hsa-miR-H6-3p	0.27
hsa-miR-335-3p	3.91	hsa-miR-4639-3p	4.31	hsa-miR-150-5p	0.27
hsa-miR-302a-3p	6.93	hsa-miR-660-5p	4.59	hsa-let-7b-5p	0.39
hsa-miR-5581-3p	2.26	hsa-miR-1827	2.74	hsa-miR-3175	0.46
hsa-miR-1260b	2.57	hsa-miR-1255-3p	8.04	hsa-miR-30e-5p	0.18
hsa-miR-4443	4.06	hsa-miR-3646	2.71	hsa-miR-423-5p	0.01
hsa-miR-17-5p	2.60	hsa-miR-660-3p	3.03	hsa-let-7 g-5p	0.21
hsa-miR-425-5p	2.29	hsa-miR-3124-3p	4.17	hsa-miR-146b-5p	0.20
hsa-miR-21-3p	3.65	hsa-miR-4795-5p	3.56	hsa-miR-101-3p	0.14
hsa-miR-574-5p	2.85	hsa-miR-4644	3.80	hsa-miR-29b-3p	0.20
hsa-miR-4279	2.86	hsa-miR-4445-5p	12.38	hsa-miR-1908-5p	0.22
hsa-miR-3941	2.71	hsa-miR-4286	2.51	hsa-miR-4468	0.15
hsa-miR-483-3p	4.50	hsa-miR-1255a	2.62	hsa-miR-23a-3p	0.15
hsa-miR-5100	2.00	hsa-miR-32-3p	2.36	hsa-miR-26b-5p	0.26
hsa-miR-4290	2.66	hsa-let-7e-5p	3.74	hsa-miR-3607-3p	0.02
hsa-miR-642b-5p	3.25	hsa-miR-4780	3.58	hsa-miR-128-3p	0.37
hsa-miR-2115-3p	4.31	hsa-miR-5681b	3.83	hsa-miR-4299	0.02
hsa-miR-3667-5p	2.95	hsa-miR-4284	2.38	hsa-miR-221-3p	0.16
hsa-miR-4695-3p	3.45	hsa-miR-223-3p	4.05	hsa-miR-423-3p	0.38
hsa-miR-3136-3p	2.76	hsa-miR-664a-5p	38.81	hsa-miR-4750-5p	0.13
hsa-miR-5684	3.07	hsa-miR-22-5p	2.50	hsa-miR-590-5p	0.10
hsa-miR-4456	4.08	hsa-miR-21-5p	2.33	hsa-miR-342-3p	0.13
hsa-miR-4531	3.74	hsa-miR-454-3p	3.15	hsa-miR-186-5p	0.44
hsa-miR-532-5p	10.81	hsa-miR-4473	21.93	hsa-miR-25-5p	0.09
hsa-miR-513a-5p	3.88	hsa-miR-1587	2.35	hsa-miR-222-3p	0.00
hsa-miR-155-5p	2.54	hsa-miR-5187-3p	4.63	hsa-miR-5089-5p	0.02
hsa-miR-548an	2.57	hsa-miR-181b-5p	2.03	hsa-miR-126-3p	0.19
hsa-miR-4787-5p	2.02	hsa-miR-554	6.38	hsa-miR-16-5p	0.48
hsa-miR-433-5p	2.65	hsa-miR-20a-5p	2.00	hsa-miR-4777-5p	0.17
hsa-miR-1264	4.51	hsa-miR-491-3p	3.90	hsa-miR-451a	0.27
hsa-miR-505-3p	2.22	hsa-miR-5002-5p	3.41	hsa-miR-339-5p	0.17
hsa-miR-2116-5p	8.23	hsa-miR-891a-3p	2.74	hsa-miR-146a-5p	0.04
hsa-miR-30e-3p	2.03	hsa-miR-4421	2.26	hsa-miRPlus-A1015	0.44
hsa-miR-4423-5p	5.95	hsa-miR-199b-5p	15.95	hsa-miR-374b-5p	0.18
hsa-miR-4742-3p	5.87	hsa-miR-191-5p	2.62	hsa-miR-378a-3p	0.45
hsa-miR-205-3p	6.12	hsa-miR-365a-3p	3.29	hsa-miR-3653-3p	0.12
hsa-miR-223-5p	17.44	hsa-miR-106a-5p	2.56	hsa-miR-451b	0.23
hsa-miR-27a-5p	5.92	hsa-miR-3182	4.77	hsa-miR-28-5p	0.35
hsa-miR-2355-3p	4.63	hsa-miR-3149	2.81	hsa-miR-361-3p	0.41
hsa-miR-378c	3.90	hsa-miR-1273 g-3p	5.39		
hsa-miR-620	10.31	hsa-miR-23b-3p	2.25		
hsa-miR-1246	6.10	hsa-miR-4454	2.75		

### B7-H3 is the target of miR-29c confirmed by luciferase reporter assay

**Fig. 2:**
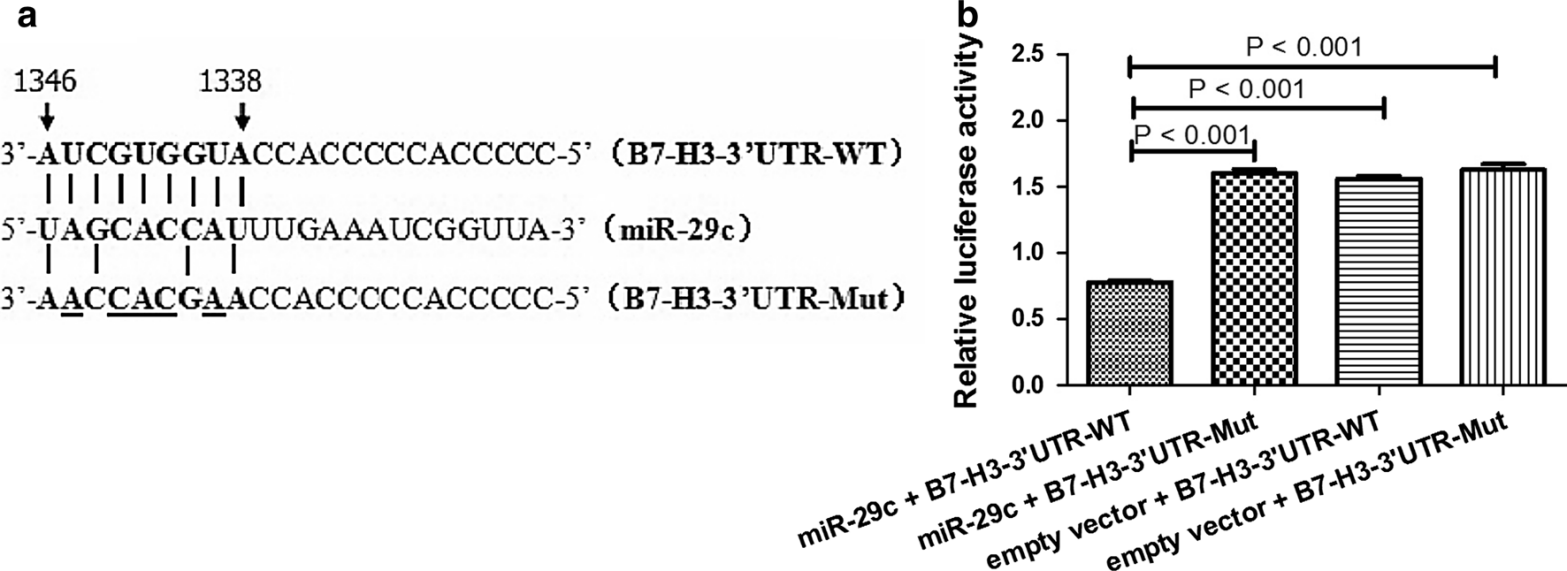
B7-H3 is the target of miR-29c confirmed by luciferase reporter assay. **a** The binding site of miR-29c in the 3′UTR of the B7-H3 mRNA and the mutant sequence of 3′UTR of B7-H3 mRNA. **b** Luciferase activity in THP-1 cells transiently co-transfected with pLenti-miR-29c or empty vector and B7-H3-3′UTR reporter plasmid or B7-H3-3′UTR-Mut reporter plasmid. Data are shown as mean ± STD

As shown in Fig. 2a, miR-29c targets wide-type B7-H3-3′untranslated region (3′UTR) and the mutated sequences of B7-H3-3′UTR. To determine if miR-29c acts directly on B7-H3 expression, a luciferase reporter assay was performed. The targeting site was cloned into the 3′UTR of the firefly luciferase gene and co-transfected with miR-29c into THP-1 cells. Co-transfection group of B7-H3-3′UTR-WT and miR-29c significantly reduced luciferase activity compared to B7-H3-3′UTR-WT group and control group (empty vector), reduced by 50% as shown in Fig. 2b. However, this repression was not shown while THP-1 cells transfected with some mutations in binding site of B7-H3-3′UTR (Fig. 2b).

### Regulation of B7-H3 expression by miR-29c on THP-1 cells

**Fig. 3:**
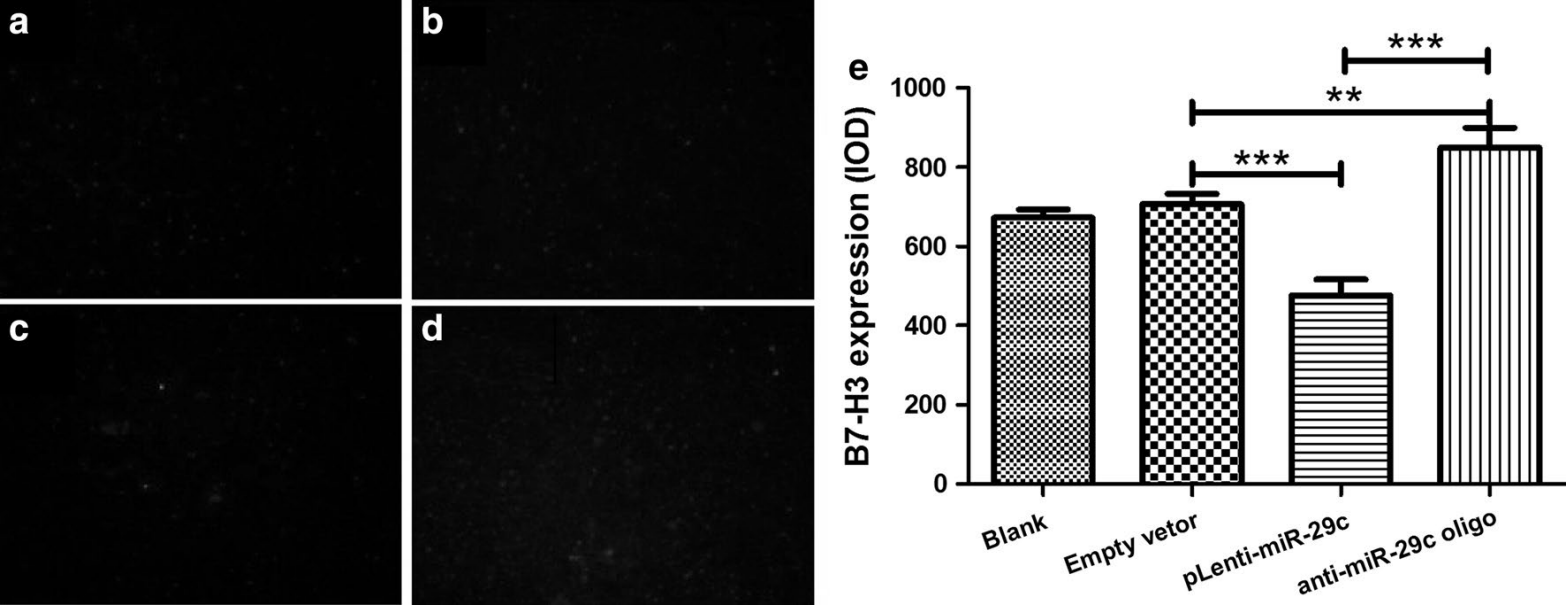
Regulation of B7-H3 expression by miR-29c on THP-1 cells. pLenti-miR-29c or anti-miR-29c oligo was transfected into THP-1 cells the expression of B7-H3 was detected by immunofluorescence staining. **a** Blank control. **b** Empty vector. **c** pLenti-miR-29c. **d** Anti-miR-29c oligo. **e** Comparison of grey levels of B7-H3 expression using Image-Pro Plus software. **P < 0.01; ***P < 0.001. Data are shown as mean ± STD

To investigate the possible impact of miR-29c on B7-H3, pLenti-miR-29c was transfected into THP-1 cells and the expression of B7-H3 was detected by immunofluorescence staining. As shown in Fig. 3a, b, up-regulation of miR-29c significantly decreased B7-H3 expression compared to negative control (empty vector), while down-regulation of miR-29c significantly increased B7-H3.

### Function of miR-29c on macrophage in regulating Th cell differentiation

**Fig. 4:**
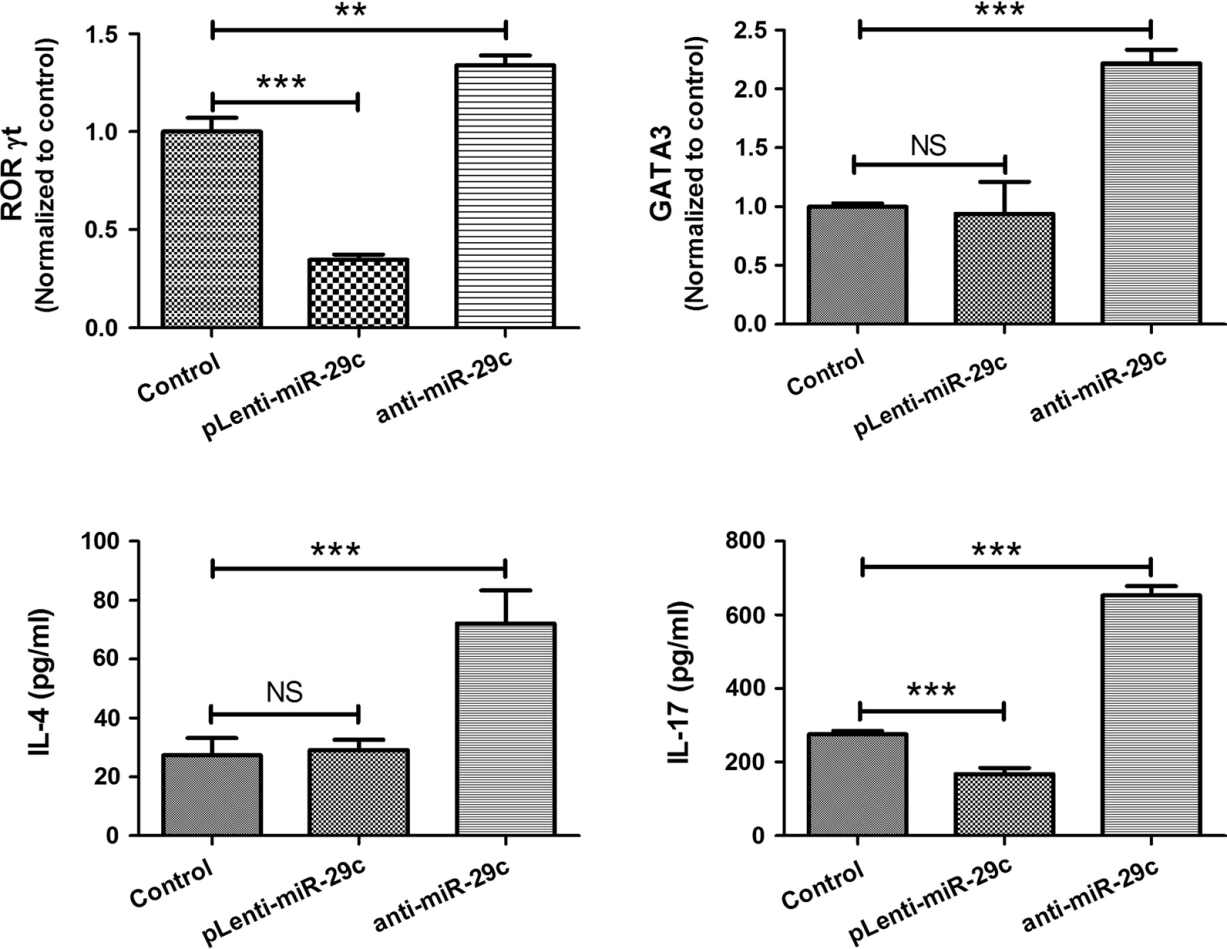
Function of miR-29c on macrophage in regulating Th cell differentiation. ROR-γt and GATA-3 expression were increased in CD4^+^ T cells compared to control group when CD4^+^ T cells co-cultured with macrophage transfected with anti-miR-29c. ROR-γt expression was decreased when CD4^+^ T cells co-cultured with macrophage transfected with pLenti-miR-29c. Levels of IL-4 and IL-17 in supernatants were increased in anti-miR-29c group and IL-17 was decreased in pLenti-miR-29c group. *NS* not significant; **P < 0.01; ***P < 0.001. Data are shown as mean ± STD

To investigate the role of miR-29c in regulating Th cell differentiation, macrophage transfected with pLenti-miR-29c or anti-miR-29c was co-cultured with CD4^+^ T cells. Then, proliferated T cells were harvested and cultured for another 24 h. As shown in Fig. 4, ROR-γt and GATA-3 expression were increased in CD4^+^ T cells compared to control group when CD4^+^ T cells co-cultured with macrophage transfected with anti-miR-29c (P < 0.01). However, Only ROR-γt expression was decreased compared to control group when CD4^+^ T cells co-cultured with macrophage transfected with pLenti-miR-29c (P < 0.001). With the same trend, levels of IL-4 and IL-17 in supernatants were increased in anti-miR-29c group (both P < 0.01) and only IL-17 was decreased in pLenti-miR-29c group (P < 0.001).

### Anti-B7-H3 mAb suppresses Th cell differentiation co-cultured with macrophages transfected by anti-miR-29c

**Fig. 5:**
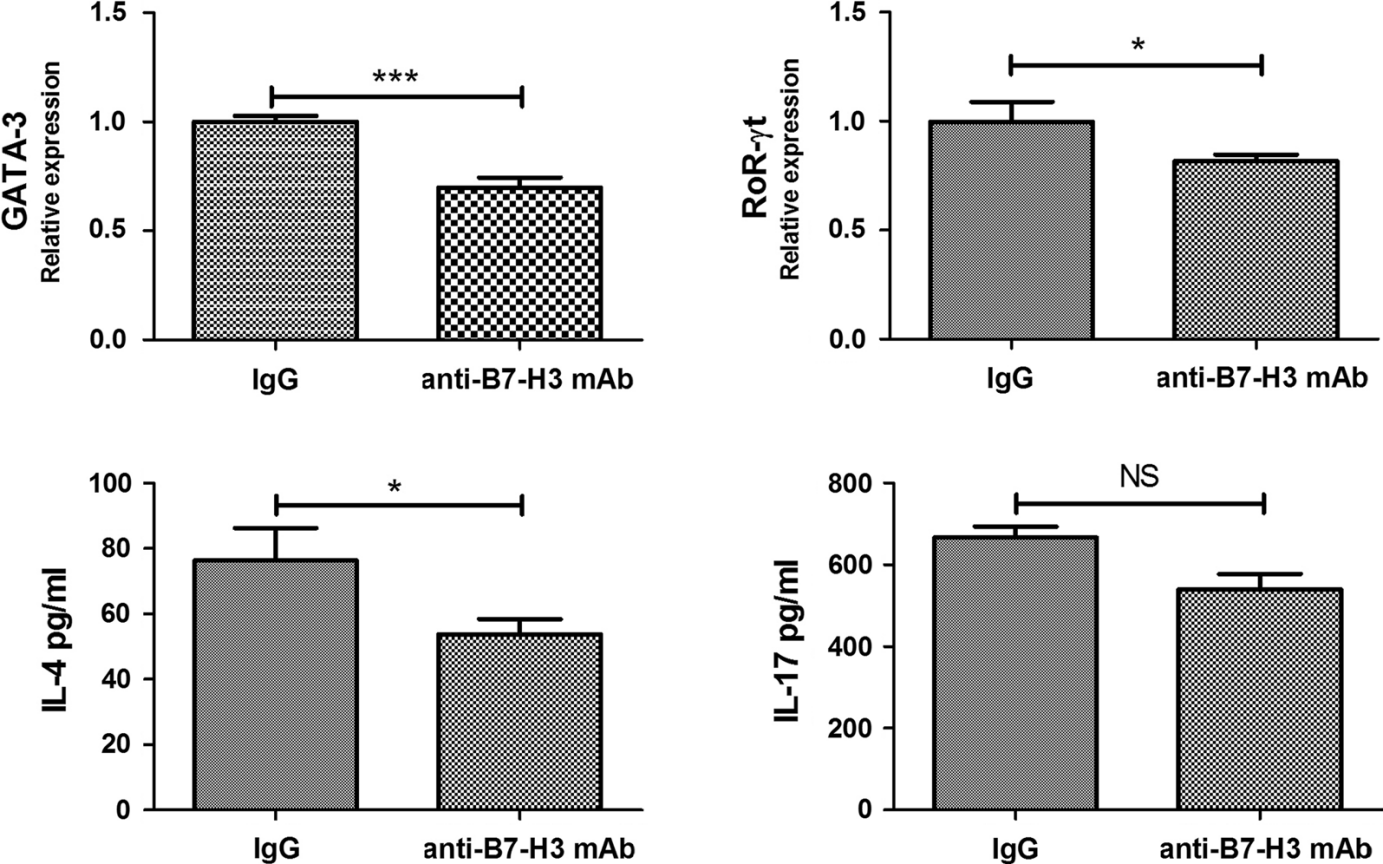
Anti-B7-H3 mAb suppresses Th cell differentiation co-cultured with macrophages transfected by anti-miR-29c. Macrophages transfected with anti-miR-29c were harvested and were co-cultured with CD4^+^ T cells (1 × 10^5^ cells/ml) in the presence of control IgG (20 µg/ml) or anti-B7-H3 mAbs (20 µg/ml). ROR-γt and GATA-3 expression were decreased in CD4^+^ T cells compared to control IgG group. The level of IL-4 in supernatant was also decreased compared to control IgG group. However, an addition of anti-B7-H3 in the cultures had no effect on expression of IL-17. *NS* not significant; *P < 0.01; ***P < 0.001. Data are shown as mean ± STD

To further evaluate whether anti-miR-29c suppresses Th cell differentiation through enhancing the expression of B7-H3, macrophages were transfected by anti-miR-29c and then anti-B7-H3 mAb was added into cultures of macrophages and CD4^+^ T cells. As shown in Fig. 5, ROR-γt and GATA-3 expression were decreased in CD4^+^ T cells compared to control IgG group (both P < 0.05). The level of IL-4 in supernatant was also decreased compared to control IgG group (P < 0.05). However, an addition of anti-B7-H3 in the cultures had no effect on expression of IL-17 (P > 0.05). Taken together, our present results suggest that miR-29c/B7-H3 axis plays an important role in asthma, particularly Th cell differentiation.

### Evaluation of miR-29c in monocytes and plasma B7-H3 in asthmatic children and controls

**Fig. 6:**
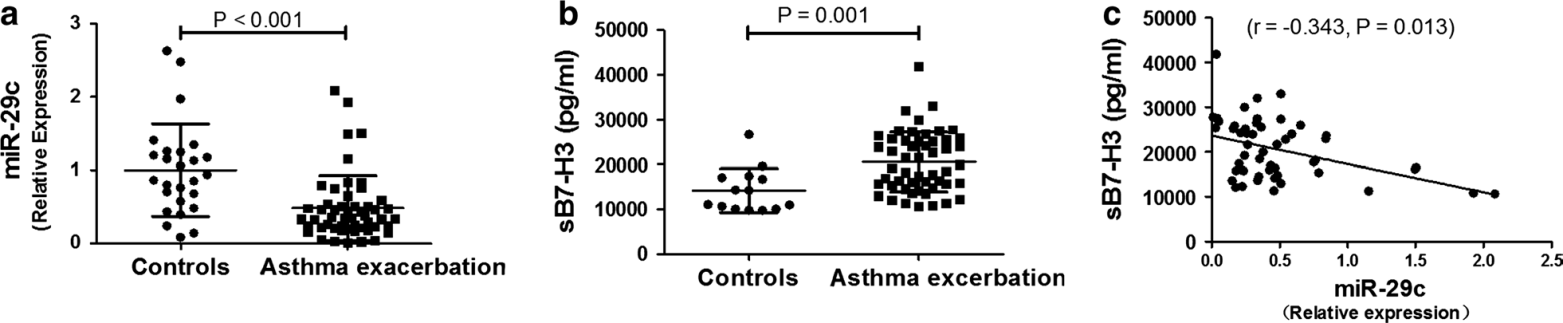
Evaluation of miR-29c and plasma B7-H3 in hospitalized asthmatic children and controls. **a** Comparison of miR-29c between asthmatic children and controls. **b** Comparison of plasma B7-H3 between asthmatic children and controls. **c** Correlation between miR-29c and plasma B7-H3 of asthmatic children

According to TargetScan 7.1 database, B7-H3 is the direct target of miR-29c. To determine the association between miR-29c and B7-H3 in asthmatic children, qPCR and ELISA were used for miR-29c and plasma B7-H3 detection, respectively. As shown in Fig. 6a, children with asthma exacerbation had significantly lower level of miR-29c in monocytes compared to controls (0.48 ± 0.44 vs. 1.00 ± 0.63, relative expression) and higher level of plasma B7-H3 (20,594 ± 6706 vs. 14,180 ± 4920 pg/ml) shown in Fig. 6b. Meanwhile, miR29c concentration was negatively associated with plasma B7-H3 concentration (r = − 0.343, P = 0.013, Fig. 6c). There were no differences of miR-29c and plasma B7-H3 between mild to moderate asthmatics and severe asthmatics (both P > 0.05).

### Levels of miR-29c and plasma B7-H3 on admission and discharge

**Fig. 7:**
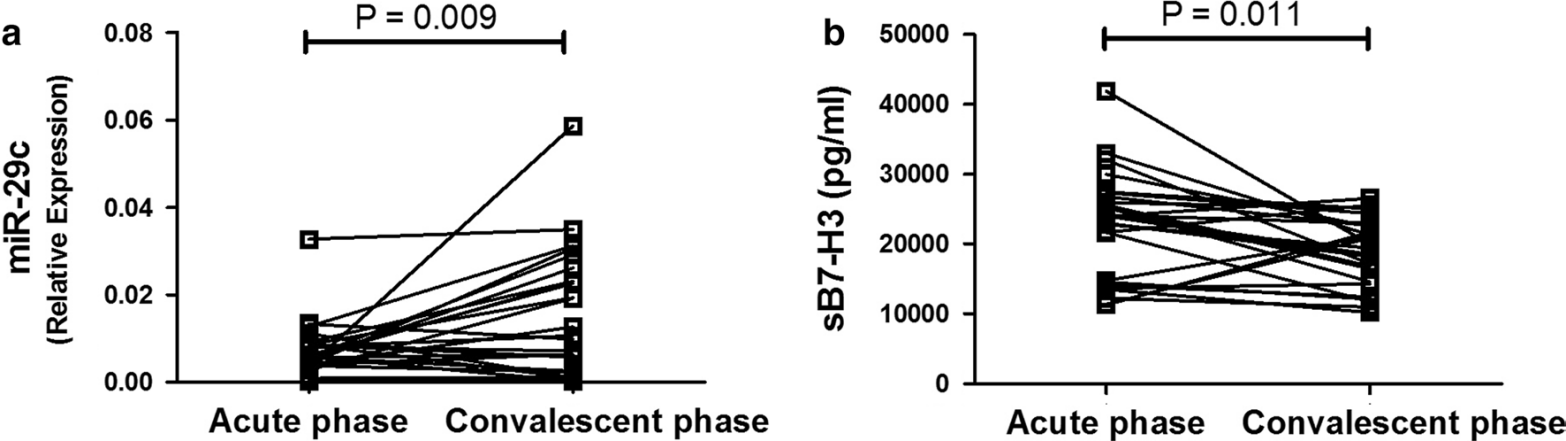
Levels of miR-29c and plasma B7-H3 on acute phase and convalescent phase. **a** Level of miR-29c significantly increased in convalescent phase compared to that of acute phase. **b** Level of plasma B7-H3 significantly decreased in convalescent phase compared to that of acute phase

Of all asthmatic children, 27 subjects also were detected miR-29c and plasma B7-H3 on discharge. Level of miR-29c significantly increased during convalescent phase compared to that of acute phase (P = 0.009, Fig. 7a) while plasma B7-H3 significantly decreased during convalescent phase (P = 0.011, Fig. 7b).

## Discussion

Current therapy for mild or moderate asthma is based on inhaled corticosteroids in combination with inhaled long-acting beta-agonists or oral leukotriene receptor antagonist. Although current therapy is generally satisfactory, a minority of children has persistent uncontrolled asthma and frequently exacerbations [19]. It has been suggested that miRNAs may be useful as biomarkers of bronchial asthma [20]. In this study, we demonstrated that miR-29c plays a vital role in the development of asthma exacerbation through targeting costimulatory molecule B7-H3.

Of all differentially expressed miRNAs in peripheral blood CD14^+^ monocytes, 88 miRNAs were up-regulated and 41 miRNAs down-regulated in asthma children compared to controls. A recent similar study showed that 36 miRNAs were significantly upregulated and 47 significantly downregulated 2-fold in the asthmatic group compared to controls [21]. But this study was focused on lymphocytes not the monocytes in peripheral blood. A previous study [22] indicated that miRNAs are not involved in the development of allergic asthma by miRNA array analysis. However, that study enrolled mild asthma patients without exacerbation and increasing evidence suggests that miRNAs could regulate biological processes including T cell differentiation during the course of asthma development [11–13].

Although no study reports the pathogenesis of miR-29c in asthma, a recent study demonstrated that miR-29c could regulates the expression of matrix metalloproteinase-2 (MMP-2) in primary rat aorta smooth muscle cells and MMP-2 is the target of miR-29c confirmed by luciferase reporter activity assay. Furthermore, glucocorticoids time-dependently increased miR-29c expression [23]. Meanwhile, MMP-2 is one of main mediators in the pathogenesis of airway remodeling in asthma [24, 25]. Taken together, we presume that miR-29c expression induced by glucocorticoids might play important role in alleviating asthma through miR-29c targets such as MMP-2.

In present study, it is confirmed that B7-H3 was another target of miR-29c and miR-29c maybe take parts in the development of asthma through regulating B7-H3 expression. Similar to the present study, several previous studies reported that miR-29c was inversely correlated to its target gene B7-H3 [14, 15, 26]. B7-H3 could enhance cell migration and invasion in various tumors including renal cell carcinoma [14], cutaneous melanoma [15], and breast cancer [26]. Besides tumors, B7-H3 also takes parts in inflammatory diseases such as sepsis [27] and bacterial meningitis [28]. Our previous studies also demonstrated that B7-H3 plays an important role in the development of asthma by augmentation of the inflammatory response [5, 6].

In summary, our study demonstrated that low expression of miR-29c and high expression of B7-H3 exist in children with asthma exacerbation and miR-29c expressed on macrophages could regulate Th cell differentiation through targeting B7-H3. MiR-29c has the potential to be a biomarker and provide the new approach of therapy for asthma.

## Abbreviations

AHR: hyper-responsiveness
Th2: T-helper type 2
APC: antigen presenting cell
IL: interleukin
miRNA: microRNA
